# The Role of Intestinal Fungi in the Pathogenesis and Treatment of Ulcerative Colitis

**DOI:** 10.3390/microorganisms13040794

**Published:** 2025-03-31

**Authors:** Yujing Zhang, Lin Wang, Lihua Peng

**Affiliations:** 1 Microbiota Laboratory, Clinical Division of Microbiota, Department of Gastroenterology and Hepatology, The First Medical Center of Chinese PLA General Hospital, Beijing 100853, China; zhangyujing1013@163.com (Y.Z.); wanglin1992119@126.com (L.W.); 2 Medical School of Chinese PLA, Beijing 100853, China

**Keywords:** ulcerative colitis, intestinal fungi, Candida, FMT introduction

## Abstract

Ulcerative colitis (UC) is a chronic inflammatory bowel disease closely associated with dysbiosis of the gut microbiome, encompassing not only bacterial communities but also fungal populations. Despite the growing recognition of the gut microbiome’s role in UC pathogenesis, the contribution of intestinal fungi has only recently garnered significant attention. In this review, we comprehensively examine the characteristics of intestinal fungi in both healthy individuals and UC patients, elucidating their role in disease pathogenesis and their interactions with bacterial communities. Additionally, we explore the impact of intestinal fungi on disease severity and therapeutic responses in UC. Furthermore, we evaluate the therapeutic potential of antifungal agents, probiotics, and fecal microbiota transplantation (FMT) in UC management, emphasizing the critical role of fungi in these treatment modalities. Future research should prioritize elucidating the multifunctional roles of fungi in UC pathogenesis and their implications for treatment strategies. Moreover, the identification of fungal biomarkers associated with FMT efficacy could pave the way for precision medicine approaches in FMT, offering novel insights into personalized therapeutic interventions for UC.

## 1. Introduction

Ulcerative colitis (UC), a chronic inflammatory bowel disease (IBD), is characterized by recurrent episodes and treatment resistance, significantly impairing patients’ quality of life and imposing a substantial economic burden on healthcare systems [1]. The clinical manifestations of UC can be categorized into gastrointestinal symptoms and extraintestinal manifestations. Gastrointestinal symptoms typically present as diarrhea, abdominal pain, and mucoempulent blood stool. Extraintestinal manifestations commonly include primary sclerosing cholangitis and peripheral arthritis, among others [2]. Current therapeutic interventions for UC primarily consist of: 5-aminosalicylic acid, sulfasalazine, corticosteroids, immunosuppressants, and biologics [2]. The pathogenesis of UC is widely attributed to gene–environment interactions, which trigger an aberrant immune response to the intestinal microbiota [3]. While the role of gut bacteria in UC has been extensively investigated [4,5], the contribution of gut fungi has only recently emerged as a critical area of research. The fungal microbiome is an integral part of the gut microbiota, interacting with the host immune system and influencing physiological functions [6,7], and is closely related to the development and prognosis of UC, which is characterized by an imbalance in the intestinal microecology [8]. Studies suggest that fungal microbiota are associated with disease activity in UC [9] and that fungal microbiota signatures may serve as predictive biomarkers of therapeutic response to infliximab in patients with IBD [10]. Notably, intricate interactions exist between intestinal fungi and bacteria [11], yet the mechanisms underlying their cross-talk and its impact on UC pathogenesis remain poorly understood and warrant further exploration.

In recent years, rapid advancements have been made in therapeutic approaches targeting the gut microbiome, including probiotics, prebiotics, and fecal microbiota transplantation (FMT). Unlike single- or multistrain probiotic therapies, FMT involves the transfer of a complex microbial community, including bacteria, fungi, viruses, and their metabolites, from healthy donor stool to recipients. FMT has demonstrated broad therapeutic potential across a range of diseases, such as *Clostridium difficile* infection (CDI), inflammatory bowel disease (IBD), irritable bowel syndrome (IBS), and obesity [12]. However, the clinical response to FMT in IBD patients, especially those with UC, exhibits considerable heterogeneity [13,14,15], making it challenging to predict clinical efficacy. Studies have shown that gut fungi significantly influence the therapeutic effects of FMT [16,17]. For example, a reduction in *Candida* after FMT has been associated with an improvement in the severity of the disease [18]. Therefore, there is an urgent need to explore the specific mechanisms of intestinal fungi in FMT and to search for fungal biomarkers associated with FMT efficacy.

In this review, we discuss the characterization of fungal communities in healthy individuals and UC patients, the role of fungi in the pathogenesis of UC, and the impact of their interactions with bacteria on UC. We also summarize the therapeutic effects of antifungal drugs, probiotics, and FMT in ulcerative colitis, emphasizing the important role of gut fungi in these treatment modalities.

## 2. Overview of the Gut Mycobiota

### 2.1. Types and Distribution of Intestinal Fungi

Fungi are generally considered a relatively minor component of the gut microbiota, accounting for approximately 0.1% of the microbiome in healthy populations [19]. Fungi are categorized into sexual and asexual forms, which may influence their annotation and limit research on them. The number of gut fungi increases sequentially from the ileum to the colon, peaking in the distal colon [20]. A comparison of intestinal luminal and mucosal fungi reveals that the alpha diversity (a metric of microbial community richness and evenness) of the former is higher than that of the latter [21,22]. This discrepancy can be attributed to the influence of environmental factors on fecal fungi, as well as the transient presence of certain fungi in the gut lumen.

The diversity of the gut mycobiome is comparatively lower than that of gut bacteria, with the mycobiome predominantly consisting of yeasts, which are principally from the phyla *Ascomycota* and *Basidiomycota* [23,24]. At the genus level, the gut mycobiota of healthy adults is predominantly characterized by *Saccharomyces*, *Malassezia*, and *Candida* [23]. A comprehensive fungal analysis of 3363 stool samples from populations in Europe, North America, and Asia similarly confirmed that *Saccharomyces* and *Candida* are the most abundant genera across all samples, followed by *Penicillium* and *Aspergillus* [25], whereas *Malassezia* was less prevalent in these studies, potentially due to geographical and methodological variations. In healthy older adults (aged > 65 years), the gut mycobiome is primarily dominated by *Penicillium*, *Candida*, *Saccharomyces*, and *Aspergillus* [26]. Among these, *Candida* consistently emerged as the dominant genus across multiple studies [26]. The most commonly identified *Candida* species in the human gut include *Candida albicans*, *Candida glabrata*, *Candida dubliniensis* and *Candida parapsilosis*, while *Candida tropicalis* is more prevalent in murine models [27].

A core mycobiome may exist within the human gut, as evidenced by studies demonstrating consistent fungal taxa across individuals. In the Human Microbiome Project (HMP) cohort study, gut fungal communities exhibited significant inter-individual variability and longitudinal heterogeneity. However, three fungal species—*Saccharomyces cerevisiae*, *Saccharomyces limpetus*, and *Candida albicans*—were detected in more than 60% of the samples, suggesting their potential role as core members of the gut mycobiome [23]. This finding is further supported by a longitudinal study involving 184 healthy participants, which revealed that 11 genera from the *Ascomycota* phylum, including *Pichia*, *Alternaria*, and *Wickerhamiella*, demonstrated remarkable stability over a 3.2-year follow-up period [28].

Based on taxonomic profiling, gut fungal communities can be classified into four distinct enterotypes: Saccharomyces_type, dominated by *Saccharomyces cerevisiae*; Candida_type, dominated by *Candida albicans*; Aspergillus_type; and mixed Asc_type, which reflects structural variations in fungal colonies due to differences in the amplification regions of ITS1 and ITS2 [25]. Different sequencing technologies for fungi may have biases between sequencing results due to differences in amplification fragmentation, specificity, and accuracy of the primers used, which can lead to different results in subsequent analyses [29]. Notably, the gut fungal community exhibits strong functional synergy with the fecal metabolome [28]. Despite the high taxonomic variability, the metabolic functions of the gut mycobiome are relatively conserved and exhibit low diversity. This functional redundancy, observed across multiple phyla and over time, suggests that defining the gut microbiota based on metabolic functions—rather than taxonomy—may provide a more robust framework for understanding its role in host physiology [30]. Such functional conservation is further supported by the identification of inferred metabolic modules derived from proteomic data, as mapped to KEGG homolog groups [30].

### 2.2. Factors Affecting Intestinal Fungal Communities

Diet is one of the main factors influencing intestinal fungal colonization [31,32,33]. *Saccharomyces cerevisiae*, a species commonly present in fermented foods and bread, is often one of the most abundant species in the human mycobiome. Moreover, carbohydrate-rich diets have been shown to positively correlate with the abundance of intestinal *Candida*, whereas high-protein diets are inversely associated with the levels of *Methanobrevibacter* and *Candida* populations in healthy volunteers [34]. Further studies have indicated that high levels of animal protein can exacerbate dextran sulfate solution (DSS)-induced colitis in mice by promoting proinflammatory responses in monocytes, but high plant protein does not [35]. Vitamins A and D exhibit antifungal properties, with vitamin D3 being considered a “good antifungal therapy” [36].

The composition of human gut fungal communities exhibits significant variation across geographic locations [25,28]. A comparative study on the distribution of gut fungi among residents of Hong Kong and Yunnan revealed pronounced differences, with a ratio of 15:2 in the enrichment of different fungal species in the feces of Yunnan and Hong Kong residents, and similar results were obtained in the validation cohort [37]. Intestinal fungi are influenced not only by the above factors but also by various other elements. Age is correlated with certain fungal genera; for example, *Candida* is positively correlated with older individuals, whereas the genera *Saccharomyces* and *Aspergillus* are preferentially enriched in younger cohorts [25]. In contrast, body mass index (BMI) and sex are not correlated with the distribution of fungal enterotypes [25]. However, the functional composition of gut bacteria correlates with host BMI and sex. For example, the ATPase complex for energy acquisition is associated with obesity, and specific bacteria linked to aspartic acid biosynthesis are more abundant in males [38]. *Saccharomyces* may be beneficial for human metabolic health, as its relative abundance is negatively correlated with fasting blood glucose and positively correlated with high-density lipoprotein cholesterol [28].

## 3. The Role of Intestinal Fungi in the Pathogenesis of Ulcerative Colitis

### 3.1. Impact of Fungi on the Intestinal Mucosal Barrier

The intestinal mucosal barrier functions as a critical protective shield against microbial invasion, comprising three primary components: the mucus layer, epithelial cells, and intestinal immune cells. First, the mucus layer is formed by goblet cells that secrete two layers of mucus, with mucin 2 as its main component, and it also contains secretory IgA (sIgA) and other proteins, such as antimicrobial peptides [39,40]. In active ulcerative colitis, the mucus layer is thinner and more permeable [41,42]. This compromised barrier facilitates fungal interactions with epithelial cells, thereby initiating immune responses and exacerbating intestinal inflammation. Research has shown that the filamentous form of *Candida albicans* has been strongly associated with enhanced pathogenicity, as this morphological state promotes the proliferation and migration of intestinal epithelial cells, as well as the secretion of proinflammatory cytokines such as IL-8 [43]. Furthermore, sIgA within the mucus layer preferentially targets the filamentous form of *Candida albicans*, with adhesion molecules expressed by the filaments serving as direct epitopes. A deficiency in intestinal sIgA has been linked to an increased prevalence of the filamentous form of *Candida albicans*, further highlighting its role in maintaining mucosal homeostasis [44,45]. Second, the epithelial cell barrier consists of a monolayer of tightly interconnected epithelial cells, bound by tight junctions to form a robust physical barrier. The adherence of *Candida* to intestinal epithelial cells is a critical initial step in infection, followed by invasion of this barrier by active penetration [34]. Key regulatory factors, such as HIF-1α and IL-37, have been identified as essential mediators in resisting *Candida* colonization, underscoring their protective roles in maintaining epithelial integrity [46]. For intestinal immune cells, distal colonic macrophages, called “balloon-like” protrusions (BLPs) + macrophages, extend specialized “balloon-like” protrusions into the intestinal epithelium, a unique cellular adaptation that critically prevents toxin absorption and maintains epithelial barrier integrity [47]. Additionally, gut B cells also regulate the morphology of *Candida* in the intestine, effectively modulating its pathogenic potential [44].

Changes in the structure and function of the intestinal microbiota can disrupt mucosal homeostasis and lead to an exaggerated and sustained immune response to specific microbial components [48], ultimately resulting in disease progression. Multiple analyses of fecal microbial profiles from UC patients have revealed an increase in *Candida* species, which can disrupt the intestinal mucosal barrier through various mechanisms. For instance, *Candida albicans* secretes aspartyl proteases that degrade mucin, compromising the protective mucus layer of the intestinal epithelium [43]. Similarly, *Candida tropicalis* can exacerbate disease in wild-type animals by increasing gut permeability and damaging tight junctions [49]. Moreover, the Candida-dominated microbial community is associated with the biosynthesis of hemoglobin, a key iron source for pathogenic bacteria [50]. This interaction may indirectly damage the intestinal barrier by promoting the growth of other pathogenic microorganisms [25].

Despite the association of gut fungi with disease development, certain fungi exhibit protective effects under specific conditions. A study demonstrated that mucosa-associated fungi (MUC), including *Candida albicans*, *Saccharomyces cerevisiae*, and *Saccharomycopsis fibuligera*, can reduce intestinal permeability, preventing colon damage induced by DSS and decreasing mortality rates in mice [21]. IL-22 is widely recognized as a pivotal cytokine in promoting intestinal mucosal barrier repair and maintaining gut homeostasis [51]. Mucosa-associated fungi effectively stimulate IL-22 production from CD4+ T cells, enhancing the functionality of the intestinal barrier and participating in JAK/STAT signaling and the transcription of epithelial genes involved in DNA repair, thus playing an immune-protective role in the intestine [21].

Therefore, understanding the interactions between fungi and the intestinal mucosal barrier may be crucial for elucidating the pathogenesis of intestinal diseases.

### 3.2. Intestinal Fungi and Immunity in UC Patients

The fungal cell wall is critical for fungal survival and reproduction and is a key target for antifungal drugs and the host immune system [52]. Fungal cell walls are divided into two layers, with the inner layer consisting mainly of β-glucans and chitin and the outer layer consisting mainly of a diverse range of glycoproteins. For example, the outer layers of the cell walls of *Saccharomyces cerevisiae* and *Candida albicans* are enriched in mannose glycosylated glycoproteins [53]. In response to fungal invasion, various host receptors can initiate an immune response by recognizing components of the fungal cell wall. Genetic evidence and experimental studies highlight the central role of C-type lectin receptors (CLRs) in antifungal immunity, whereas Toll-like receptors (TLRs) and nucleotide oligomerization domain (NOD)-like receptors (NLRs) generally play secondary roles [54]. CLRs are expressed primarily by phagocytic cells such as macrophages and dendritic cells, with common types including Dectin-1, Dectin-2, Dectin-3, and Mincle.

**Figure 1:**
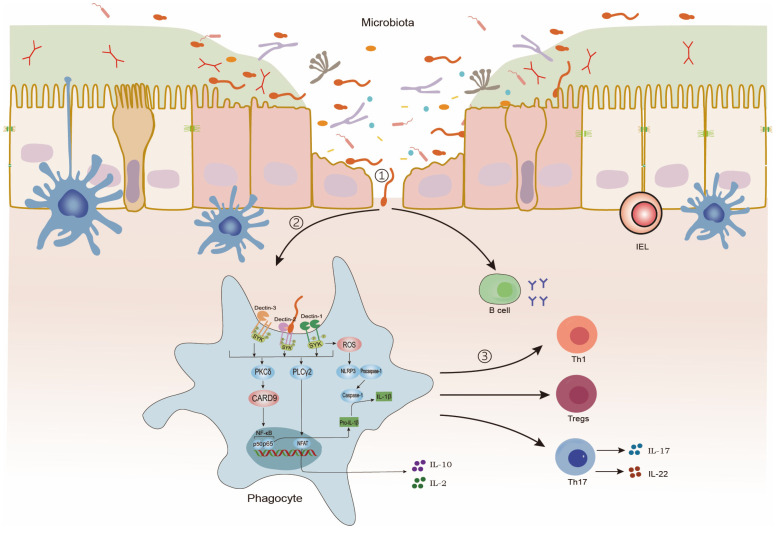
Intestinal immunity against intestinal fungi. ①: Intestinal fungi invade the intestinal epithelium. ②: Phagocytes recognize and present intestinal fungi. ③: B-cell and T-cell immune response.

Within the intestinal mucosa, CLRs recognize fungal cell wall components, triggering a series of immune responses. This interaction initiates caspase recruitment domain-containing protein 9 (CARD9)-dependent nuclear factor kappa-B (NF-κB) activation, leading to the production of IL-1β, a cytokine critical for protection against colitis. Concurrently, it promotes the secretion of cytokines such as IL-2 and IL-10. Phagocytes recognize, internalize, and process fungal antigens, subsequently directing the differentiation of CD4+ T cells into functionally distinct subsets—such as Th17, Th1, and regulatory T cells (Tregs)—via divergent intracellular signaling cascades. Unlike T cells, B cells directly recognize fungal antigens, producing immunoglobulins [7,54,55,56,57,58,59] (Figure 1).

Phagocytosis in response to fungi initiating intestinal fungal immunity plays a crucial part in the immune response. CX3CR1+ mononuclear phagocytes (CX3CR1+ MNPs) are key mediators in orchestrating the Th17 immune response against fungal infections. These cells recognize both commensal and pathogenic fungi, such as *Candida*, through CLRs [60]. Additionally, CX3CR1+ MNPs regulate early *Candida* infections by suppressing caspase-dependent apoptosis and enhancing Akt phosphorylation [34]. Moreover, CX3CR1+ MNPs facilitate the generation of antifungal IgG antibodies in serum through T-cell-dependent mechanisms and the SYK-CARD9 signaling pathway, with IgG antibodies in healthy individuals showing preferential specificity for symbiotic *Candida* [61]. Moreover, CX3CR1+ MNPs facilitate the generation of antifungal IgG antibodies in serum through T-cell-dependent mechanisms and the SYK-CARD9 signaling pathway [62], which is closely related to the impact of gut fungi on the host.

In addition to direct recognition by the host, gut-fungi-derived metabolites play a pivotal role in modulating host immune responses. *Candida albicans*, for instance, induces phagocyte lysis through both inflammasome-dependent pathways and the formation of membrane-penetrating hyphae. Furthermore, its secreted toxin, candidalysin, facilitates phagocyte dissolution via a non-inflammasome-dependent mechanism, thereby enabling immune evasion [63]. Additionally, lactate signaling contributes to immune escape by regulating β-glucan masking [64]. Candidalysin also drives the production of interleukin-1β (IL-1) [65], which exacerbates colitis through the modulation of Th17 cell differentiation. Intestinal fungi further influence immune dynamics by secreting prostaglandins (PGs) or converting exogenous arachidonic acid (AA) into PGs. These PGs suppress antifungal Th1 responses, inhibit lymphocyte proliferation, and impair phagocytosis by promoting Th2 cell responses, thereby skewing adaptive immunity to favor *Candida* survival [66,67]. Collectively, these interactions between gut fungi and the immune system are intricately linked to the pathogenesis of various diseases.

The interaction between gut fungi and the host immune system is complex and multifaceted, with different fungal species exerting varying effects on immune responses. For instance, *Saccharomyces cerevisiae*, recognized for its anti-inflammatory properties, has been shown to significantly elevate levels of the anti-inflammatory cytokine IL-10 [8]. In contrast, another study demonstrated that Saccharomyces cerevisiae exacerbates colitis without inducing a prominent T-cell inflammatory response in the gut or in vitro. This effect may be mediated through the upregulation of uric acid via purine metabolism, thereby influencing colitis progression [68].

Disruptions in any component of the immune response to intestinal fungi can predispose the host to disease development. Dectin-1, Dectin-2, Dectin-3, and CARD9 are key molecules responsible for host defense against fungi, and mutations in any of the genes encoding these molecules correlate with human susceptibility to fungal infections [27]. Furthermore, defects in these molecules exacerbate the severity of dextran sulfate sodium (DSS)-induced colitis in murine models [49,69]. Dectin-1, a receptor for fungal β-glucans [70], plays a critical role in fungal recognition. Knockout of the CLEC7A gene, which encodes Dectin-1, results in heightened susceptibility to chemically induced colitis due to altered responses to resident fungi. Additionally, polymorphisms in the Dectin-1 gene have been correlated with the severity of UC [49]. However, Tang et al. reported that the absence of Dectin-1 may provide protection against DSS-induced colitis, potentially because of the original fungal characteristics in the gut [71]. Intriguingly, dual deficiency of Dectin-1 and Dectin-2 has been shown to protect against DSS-induced colitis, with this protective effect mediated by gut bacteria rather than fungi. Specifically, the *Lachnospiraceae* family has been identified as a key protective bacterial group in this context [72]. In addition to the strong association of Dectin-1 and Dectin-2 with fungal immunity, studies have shown that Dectin-3-deficient mice are more susceptible to the induction of DSS-induced colitis and are defective in promoting tissue repair in colonic epithelial cells, possibly related to defective NF-κB activation and reduced IL-6 production [55]. Similarly, CARD9 is essential for the host immune response to fungi. CARD9 knockout in mice leads to mitochondrial dysfunction and apoptosis, which may explain the heightened susceptibility to intestinal inflammation and fungal infections observed in CARD9-deficient individuals [73].

### 3.3. The Effects of Intestinal Fungal–Bacterial Interactions on UC

Studies have shown that the positive correlation between bacteria and fungi is greater in UC patients than in healthy individuals and that the negative correlation is greater in UC patients than in those with Crohn’s disease (CD) [8]. Furthermore, prolonged antibiotic use has been shown to disrupt the microbial balance, leading to fungal overgrowth and increased susceptibility to fungal infections due to the depletion of bacterial populations [74,75]. These findings underscore the intricate interplay between fungi and bacteria, suggesting that cross-kingdom interactions play a pivotal role in disease pathogenesis [76,77]. First, one key mechanism underlying this interaction involves the modulation of the fungal colonization environment by bacterial metabolites. Short-chain fatty acids (SCFAs), which are primarily produced by gut bacteria, are crucial for maintaining intestinal homeostasis and mucosal immunity [78,79]. Some studies have shown that SCFAs possess antifungal properties and that SCFA levels are reduced in the intestines of UC patients [46,80,81], potentially facilitating changes in the gut fungal community. Supporting this, a large-scale analysis of gut fungal communities in 1244 participants with an average age of 64.9 years revealed that *Saccharomyces* bacteria exhibited a positive association with SCFA-producing bacteria, such as *Clostridium sensu stricto 1*, *Faecalitalea*, and *Megamonas* [28], whereas *Candida* bacteria were negatively correlated with major SCFA-producing gut bacteria [24,81]. Second, bacterial communities influence the immune landscape, which in turn regulates fungal survival and colonization. Bacterium-induced immune responses can limit *Candida* colonization of the gut [46]. Conversely, fungi can also impact bacteria. For example, *Bacteroides* produce specialized enzymes that degrade the mannan present on the surface of *Candida*, thereby facilitating their own proliferation through nutrient acquisition [82].

From the perspective of fungal diversity, UC patients with a relatively high baseline abundance of *Candida* exhibit increased bacterial alpha diversity, which persists for eight weeks after FMT [83]. This observation may be attributed to alterations in the bacterial production environment, which create a favorable ecological niche for *Candida albicans* colonization. Additionally, Crohn’s disease (CD) is characterized by an elevated ratio of fungal-to-bacterial diversity [8], but the specific dynamics in UC remain to be further elucidated. At the species level, *Candida* are the most extensively studied opportunistic pathogens within the gut fungal community of UC patients. The relative abundance of *Candida* in fecal samples from UC patients shows a positive correlation with *Parabacteroides diastonis* and a negative correlation with *Eubacterium hallii* and *Bifidobacterium adolescentis* [9]. Further stratification of UC patients into active and remission phases revealed that *Candida* is positively associated with *P. diastonis*, *Faecalibacterium prausnitzii*, and *Bacteroides dorei* during remission, whereas no significant bacterial correlations were observed during active phases [9]. The interplay between fungi and bacteria has been corroborated not only in clinical studies but also in murine models of colitis. The colonization of germ-free mice with specific fungi alone is insufficient to induce significant colitis under dextran sulfate sodium (DSS) challenge; however, co-colonization with specific fungi and bacteria exacerbates colitis severity [76,84].

Both synergistic and competitive interactions exist between fungi and bacteria. Research indicates that the detrimental effects of *Candida* albicans on colitis, as well as the beneficial effects of *Saccharomyces boulardii*, are contingent upon the presence of mucin-sensitive bacteria, particularly those from the *Enterobacteriaceae*, which can sustain fungal loads in the gut [76]. Conversely, competition between fungi and bacteria is also evident. In adult mice, commensal bacteria, especially those from the *Bacteroidetes* and *Firmicutes*, play a critical role in limiting *Candida* colonization [46]. For example, *Lactobacillus rhamnosus* can deplete essential nutrients (nitrogen, carbon, etc.) required by *Candida*, leading to alterations in its gene expression and metabolic activity, thereby reducing its pathogenicity [85]. Additionally, *Faecalibacterium prausnitzii* can inhibit *Candida albicans* reproduction, colonization, and pathogenicity to improve DSS-induced colitis, whereas *Escherichia coli* primarily suppresses *Candida albicans* quantity without affecting its virulence [86]. Furthermore, bacteria can modulate the morphological transition of *Candida albicans* between yeast and hyphal forms [87,88,89], thereby influencing its virulence. The reciprocal interactions between gut fungi and bacteria are both causative and dynamic, contributing to the ecological dysbiosis of intestinal microbial communities. The dual roles of fungi and bacteria in these interactions underscore their collective impact on disease progression.

## 4. Influence of Intestinal Fungi on the Severity and Treatment Response of UC

### 4.1. Characteristics of Intestinal Fungi in UC Patients

In terms of intestinal fungal diversity, the alpha diversity of fungi in the feces of UC patients is lower than that in healthy subjects [8]. However, no significant differences in fungal α-diversity or β-diversity were observed between the active and remission phases of UC [9,90]. Regarding gut fungal composition, a prospective cohort study of 421 UC patients revealed that 86% of the fungi analyzed in feces were *Ascomycetes* and 3% were *Basidiomycetes*, with *Saccharomyces* and *Candida* being the most abundant intestinal fungal genera [9]. However, another study indicated that, among IBD patients, the most abundant genus was *Candida*, followed by *Clavispora* [10]. These discrepancies may be attributed to the inclusion of CD patients in the latter study, as well as variations in disease severity among participants. Within the Ascomycota phylum, the majority of fungi belong to the Saccharomycetales order, which includes genera such as *Candida*, *Pichia*, and *Saccharomyces* [90]. At the species level, both the relative and absolute numbers of *Saccharomyces cerevisiae* in the feces of UC patients decreased, whereas the relative quantity of *Candida albicans* increased, with its absolute quantity remaining unchanged, which were unrelated to the disease phenotype [8]. However, the relative abundance of *Candida* was linked to disease activity [9,90].

Alterations in the composition of the intestinal fungal community, referred to as “fungal ecological dysbiosis”, have been implicated in the pathogenesis of both intestinal and extraintestinal disorders, including colitis, alcoholic liver disease, and hypersensitivity pneumonitis [21]. The ratio of the abundance of *Basidiomycota* to that of *Ascomycota* can represent an index of fungal ecological dysbiosis. Compared with healthy individuals, UC patients presented an increased ratio of *Ascomycota* to *Basidiomycota*, although this difference was not statistically significant [16]. Conversely, a study utilizing ITS2 sequencing of fecal fungi from 235 IBD patients and 38 healthy controls indicated that the abundance ratio of *Basidiomycota* to *Ascomycota* varied greatly among different disease phenotypes, with higher ratios during IBD flare-ups than during remission and healthy states [8].

### 4.2. Influence of Intestinal Fungi on UC Severity and Treatment Response

Dectin-1 is a host receptor that recognizes fungi. Polymorphisms in the dectin-1 gene have been linked to the severity of UC, and experimental studies have demonstrated that mice deficient in dectin-1 exhibit heightened susceptibility to colitis [49]. In murine models of colitis, the co-administration of *Candida* and DSS exacerbates disease severity, promoting systemic inflammation and intestinal dysbiosis through increased intestinal permeability [91]. Similarly, *Saccharomyces cerevisiae* also enhances intestinal permeability and aggravates colitis [68]. Clinical studies have also demonstrated a correlation between intestinal fungi and UC disease severity. The relative abundance of Candida correlates with disease severity indices, and its temporal dynamics align with the clinical activity of UC over time [9]. In patients with endoscopically active UC, elevated levels of *Saccharomyces* and *Candida* are observed, whereas *Penicillium* predominates in patients with endoscopically quiescent UC [90].

Additionally, fungi also appear to influence treatment response in UC. For instance, non-responders to infliximab therapy for IBD exhibit higher abundances of *Candida* [10]. Additionally, *Debaryomyces hansenii* has been shown to colonize injured tissue and impair mucosal healing via the myeloid cell-specific type 1 interferon-CCL5 axis, contributing to intestinal tissue damage in CD [92]. While similar mechanisms may influence tissue healing and treatment outcomes in UC, further investigation is required to elucidate these relationships.

## 5. Treatment of Ulcerative Colitis with Intestinal Fungi

### 5.1. Antifungal Drugs

Antifungal drugs, which are not conventionally used to treat IBD, are primarily utilized to manage fungal infections such as candidiasis or candidemia in immunocompromised IBD patients. Nevertheless, their therapeutic efficacy in colitis is controversial. Miconazole effectively alleviates acetic-acid-induced colitis in mice by activating nuclear factor erythroid derived 2-like 2 (Nrf2)-regulated expression of cytoprotective proteins [93]. A study was conducted in which the antifungal drug fluconazole was administered to recipient mice that had been colonized with *Candida albicans* prior to human stool infusion. This treatment resulted in the restoration of the efficacy of FMT in eradicating *Clostridium difficile* infection [94]. Conversely, Wheeler et al. reported that fluconazole disrupts intestinal fungal communities in mice, exacerbating colitis and even aggravating dust-mite-induced allergic airway disease [95]. There may be a correlation between the duration of antifungal use and the effectiveness of treatment, and the overuse of antifungals leads to gut microbial dysbiosis. While antifungal therapy reduces the prevalence of Candida, it may also promote the relative expansion of other fungal species and alter bacterial communities [95]. However, whether the observed therapeutic effects are directly mediated by changes in the fungal microbiota or indirectly through secondary impacts on bacterial populations remains unclear.

Clinical studies have demonstrated that fluconazole treatment improves both endoscopic and histological indices in UC patients with confirmed fungal infections, findings further corroborated in murine models [48]. Another study also demonstrated that fluconazole therapy enhances clinical, histological, and calreticulin levels in UC patients with *Candida* infections, with *Candida* colonization being associated with steroid use and disease activity [96].

### 5.2. Probiotics and Prebiotics

Probiotics are defined as “live microorganisms that, when administered in adequate amounts, confer health benefits to the host” [97]. Prebiotics are substrates that provide health benefits and are selectively utilized by host microbes. A randomized, double-blind, controlled trial investigating the efficacy of a combined probiotic and prebiotic treatment for UC demonstrated significant improvements in the experimental group one month posttreatment. These improvements included reduced endoscopic scores, decreased levels of human β-defensin, tumor necrosis factor-α (TNF-α), and IL-1α, and amelioration of intestinal inflammation [98]. Furthermore, a probiotic prebiotic mixture (containing *Lactobacillus casei Zhang*, *Lactobacillus plantarum P-8*, and *Bifidobacterium animalis* subsp. *lactis V9*) in combination with mesalazine reduced the UC disease activity index better than mesalazine alone and resulted in a higher remission rate [99]. A meta-analysis of 18 studies demonstrated that probiotics have significant effects on both the active and remission phases of ulcerative colitis [100]. Probiotics enhance intestinal mucosal barrier function by upregulating the expression of gut mucin-related genes, such as mucin-2 (MUC-2), and epithelial cell adhesion molecules, thereby promoting the enrichment of beneficial bacteria [101]. These alterations significantly reshape the fungal gut environment, inhibiting the growth and pathogenicity of harmful fungi.

Treatment with the probiotic formulation VSL#3 has been shown to increase the abundance and diversity of bacterial microbiota, particularly anaerobic bacteria, while simultaneously reducing fungal diversity and reinforcing intestinal epithelial barrier function [102,103]. Under TNF-α-induced intestinal mucosal injury, *Lactobacillus reuteri* has been shown to preserve the population of Lgr5+ cells, facilitate the repair of intestinal epithelial damage, attenuate the secretion of proinflammatory cytokines, and activate the Wnt/β-catenin signaling pathway through the upregulation of R-spondins, thereby promoting the proliferation of intestinal epithelial cells [104]. Additionally, *Lactobacillus rhamnosus* has been reported to mitigate disease severity in both DSS- and DSS + Candida-induced colitis models [91]. Notably, *Lactobacillus* also possesses anti-Candida properties [91,105].

Like bacteria, fungi are histoprotective and can enhance systemic and local immunity [106]. Moreover, fungal probiotics offer distinct advantages over bacterial counterparts, including inherent antibiotic resistance and the absence of antibiotic resistance gene transfer to bacterial populations [107]. *Saccharomyces cerevisiae* can effectively alleviate DSS-induced colitis through multiple mechanisms, such as reinforcing the intestinal mucosal barrier, attenuating tissue inflammation, modulating gut microbiota composition, and regulating microbial metabolism [108,109,110]. *Saccharomyces boulardii*, a member of the genus *Saccharomyces*, also has these effects [110,111,112,113]. Specifically, *Saccharomyces boulardii* activates peroxisome proliferator-activated receptor-γ (PPAR-γ) expression to prevent intestinal inflammation and IBD [114].

Despite the therapeutic potential of naturally occurring bacterial and fungal probiotics, their application is not without limitations. Consequently, engineered probiotics have attracted interest. High levels of the human P2Y2 purinergic receptor in engineered yeast increase the secretion of ATP-degrading enzymes, leading to increased breakdown of eATP, thereby limiting the eATP-driven inflammatory response, improving intestinal inflammation, and reducing intestinal fibrosis and microbial dysbiosis [115]. Similarly, engineered *Escherichia coli* strains expressing high levels of catalase and superoxide dismutase exhibit comparable effects by scavenging ROS in inflamed areas [116].

### 5.3. FMT

Currently, the primary therapeutic strategies for UC encompass 5-aminosalicylic acid, sulfasalazine, corticosteroids, immunosuppressants, and biological agents. However, these treatments are not universally effective and are often associated with adverse effects [11]. This limitation has spurred the exploration of novel therapeutic approaches. FMT has been successfully used for the treatment of CDI [117], but the clinical remission rate after FMT in patients with UC is approximately 35.0% [118]. Consequently, there is a pressing need to elucidate the key factors influencing the therapeutic efficacy of FMT to enhance its clinical outcomes in UC management.

Unlike therapies that selectively target gut bacteria, FMT addresses the entire gut microbiota, including fungi. Studies have demonstrated that targeting a healthy gut fungal community exacerbates experimental colitis in hosts with intact microbiota, which may be attributed to the protective functions performed by specific members of the gut fungal community [16]. Therefore, the therapeutic efficacy of FMT may be closely linked to gut fungi.

In studies of FMT for CDI, higher baseline relative abundances of *Penicillium* were associated with treatment failure, whereas *Dorea longicatena* and *Fecalibacillus enteris* were enriched in donors and responders to FMT [119]. Additionally, an exclusionary relationship was observed between *Dorea longicatena*, *Faecalibacillus enteris*, and *Candida* [119]. Furthermore, the abundance of *Candida albicans* in fecal samples from either FMT donors or CDI patients prior to treatment was linked to poor treatment outcomes, and eradication of *Candida albicans* from the intestinal tracts of mice via antifungal drugs prior to FMT restores the efficacy of FMT [94]. These findings support the idea that gut fungal communities play an important role in FMT treatment outcomes. In a longitudinal study of FMT-treated graft-versus-host disease (GVHD) patients, intestinal bacterial diversity increased in patients after FMT, whereas intestinal fungal diversity increased in only a few species, such as *Candida dubliniensis*, followed by a decrease in diversity [17].

Similarly, intestinal fungi play an important role in the treatment of UC via FMT. A high abundance of *Candida albicans* in the feces before FMT was associated with positive clinical findings with FMT, and a decrease in the relative abundance of *Candida* after FMT was positively associated with a decrease in disease severity. Interestingly, serum concentrations of anti-*Candida albicans* IgG remained stable in UC patients before and after FMT, whereas they increased in the control group. These observations suggest that FMT may exert its therapeutic effects, in part, by reducing *Candida* abundance and suppressing the proinflammatory immune responses triggered by fungal colonization during intestinal inflammation [16]. Another study demonstrated that encapsulated FMT improved microbial fungal diversity and composition, and patients in remission had a reduction in fungal diversity similar to that of donors. Additionally, reduced levels of pathogenic fungi, such as *Candida* and *Aspergillus hansen*, are associated with remission after encapsulated FMT in UC patients [18]. Patients who achieved remission after encapsulated FMT had specific enrichment of *Kazachstania naganishii*, *Pyricularia grisea*, *Lachancea thermotolerans*, and *Schizosaccharomyces pombe* compared with patients who did not remit [18]. *Filobasidium* is associated with clinical remission and endoscopic response after FMT in patients with mild to moderate UC, with *F. floriforme* being able to promote the release of the anti-inflammatory factor IL-10 from macrophages in vitro [120] (Table 1).

## 6. Summary and Outlook

Numerous studies have shown that the intestinal fungal community plays an important role in ulcerative colitis, intervening in various aspects of the disease through roles with the host immune system and cross-border interactions with bacteria, but many unknowns still need to be explored and added. A deeper understanding of the gut fungi of UC patients could help reveal the mechanisms of disease onset, which will guide and facilitate the precise treatment of FMT in this disease.

## Figures and Tables

**Table 1:** Correlations between intestinal fungi and FMT.

Author/Date	Details of the Study	FMT Modalities and Dosage	Donors	Therapeutic Effect	The Sequencing Technique	Changes in Intestinal Fungal Diversity After FMT	Taxonomic Changes in Intestinal Fungi After FMT	Intestinal Fungi Associated with FMT Efficacy
Haifer, C. et al./2021 [119]	Thirty-seven patients with CDI were treated with FMT, of which three received two oral FMT treatments and the rest a single FMT treatment.	Oral; 6 capsules/dose (0.35 g lyophilized feces per capsule).	There were four donors in total, the details of which are not known.	Thirty-three patients sustained clinical and biochemical cure at the end of follow-up (median follow-up time: 17 weeks).	ITS region amplicon sequencing (fITS7 and ITS4).	There were no significant differences in α-diversity as well as β-diversity between the gut fungi of responders and non-responders.	*Penicillium* differed between responders and non-responders at week one.	*Penicillium* associated with FMT failure.
Zuo, T. et al./2018 [94]	Sixteen patients with CDI received FMT and eight patients with CDI received vancomycin and were followed up for 16 weeks.	Nasoduodenal tube; 500 mL/dose.	Single donor.	At 16 weeks, 9 responders; 7 non-responders.	ITS2 sequencing.	Increased fungal abundance and diversity in FMT responders.	*Saccharomyces*, *Aspergillus*, and *Penicillum* had higher relative levels in FMT responders than in non-responders; *Candida albicans* was enriched in non-responders.	*Candida albicans* is associated with poor FMT results.
Zhang, F. et al./2021 [17]	One stage IV GvHD underwent 4 FMTs on days 0, 5, 13, and 25.	Esophagogastroduodenoscopy; 100 mL/dose.	Same single donor for the first three times, different single donor for the fourth time.	Thickening of the small intestine subsides.	Metagenomics sequencing.	Alpha diversity is reduced.	*Parastagonospora nodorum* and *Thielavia terrestris* decreased after FMT; *Saccharomyces cerevisiae*: rapid increase after 1st, decrease in 3rd; *Candida dubliniensis*: increase; *Sporisorium reilianum*: high abundance in 3rd, depleted in 4th.	None.
Leonardi, I. et al./2020 [16]	Thirty-two patients with UC underwent multidonor FMT 5 days per week, and 29 patients with UC received placebo enemas for 8 weeks.	First colonoscopic infusion, the rest are enemas; 150 mL/dose.	Three to seven unrelated donors.	Forty-four percent of the FMT group achieved steroid-free clinical remission; 20 percent of the placebo group achieved steroid-free clinical remission.	ITS1 sequencing.	Alpha diversity is reduced.	*Candida* and *Saccharomyces* were higher.	*Candida* is associated with FMT treatment.
Chen, Q. et al./2022 [18]	Twenty-two patients with active UC were treated with FMT every two days for a total of three sessions and followed up for 12 weeks.	Oral; 30 capsules/dose.	At least two donors.	At 12 weeks, 1 UC patient withdrew and 12 patients achieved clinical remission at 12 weeks (Mayo total score ≤ 2).	Metagenomic sequencing.	Alpha diversity is reduced.	*Kazachstania* and *Lachancea* were significantly enriched and the relative abundance of the genus *Ustilaginoidea* decreased, the relative abundance of *Fusarium fujikuroi* and *Candida dubliniensis* decreased, and the relative abundance of *Brettanomyces nanus*, *Kazachstania naganishii*, *Pyricularia grisea*, and *Lachancea thermotolerans* increased.	Increased relative abundance: *Pyricularia grisea*; reduced relative abundance: *Debaryomyces hansenii*, *Candida dubliniensis*, and *Candida glabrata*.

## Data Availability

No new data were created or analyzed in this study.

## Abbreviations

UC	Ulcerative colitis
FMT	Fecal microbiota transplantation
CDI	*Clostridium difficile* infection
IBD	Inflammatory bowel disease
IBS	Irritable bowel syndrome
HMP	Human Microbiome Project
DSS	Dextran sulfate solution
BMI	Body mass index
sIgA	Secretory IgA
BLPs	“Balloon-like” protrusions
MUC	Mucosa-associated fungi
CLRs	C-type lectin receptors
TLRs	Toll-like receptors
NOD	Nucleotide oligomerization domain
NLRs	NOD-like receptors
SYK	Spleen tyrosine kinase
PKCδ	Protein kinase C delta
PLCγ2	Phospholipase C gamma 2
CARD9	Caspase recruitment domain-containing protein 9
NF-κB	Nuclear factor kappa light chain enhancer of activated B cells
NFAT	Nuclear factor of activated T cells
ROS	Reactive oxygen species
CX3CR1+ MNP	CX3CR1+ mononuclear phagocytes
PG	Prostaglandin
AA	Arachidonic acid
CLEC7A	C-type lectin domain-containing 7A
CD	Crohn’s disease
SCFAs	Short-chain fatty acids
Nrf2	Nuclear factor erythroid derived 2-like 2
TNF-α	Tumor necrosis factor-α
MUC-2	Mucin-2
PPAR-γ	Peroxisome proliferator-activated receptor-γ
GVHD	Graft-versus-host disease
